# Al-Decorated C_2_N Monolayer as a Potential Catalyst for NO Reduction with CO Molecules: A DFT Investigation

**DOI:** 10.3390/molecules27185790

**Published:** 2022-09-07

**Authors:** Xinmiao Liu, Yunjie Xu, Li Sheng

**Affiliations:** MIIT Key Laboratory of Critical Materials Technology for New Energy Conversion and Storage, School of Chemistry and Chemical Engineering, Harbin Institute of Technology, Harbin 150001, China

**Keywords:** NO catalytic reduction, C_2_N monolayer, Al-C_2_N catalyst, nitric oxide, DFT calculation

## Abstract

Developing efficient and economical catalysts for NO reduction is of great interest. Herein, the catalytic reduction of NO molecules on an Al-decorated C_2_N monolayer (Al-C_2_N) is systematically investigated using density functional theory (DFT) calculations. Our results reveal that the Al-C_2_N catalyst is highly selective for NO, more so than CO, according to the values of the adsorption energy and charge transfer. The NO reduction reaction more preferably undergoes the (NO)_2_ dimer reduction process instead of the NO direct decomposition process. For the (NO)_2_ dimer reduction process, two NO molecules initially co-adsorb to form (NO)_2_ dimers, followed by decomposition into N_2_O and O_ads_ species. On this basis, five kinds of (NO)_2_ dimer structures that initiate four reaction paths are explored on the Al-C_2_N surface. Particularly, the cis-(NO)_2_ dimer structures (D_cis-N_ and D_cis-O_) are crucial intermediates for NO reduction, where the max energy barrier along the energetically most favorable pathway (path II) is as low as 3.6 kcal/mol. The remaining O_ads_ species on Al-C_2_N are then easily reduced with CO molecules, being beneficial for a new catalytic cycle. These results, combined with its low-cost nature, render Al-C_2_N a promising catalyst for NO reduction under mild conditions.

## 1. Introduction

The increasing emission of nitrogen oxides (NO_x_) has brought serious harm to the atmospheric environment and human health [1,2,3]. Nitric oxide (NO), which comprises approximately 95% of NO_x_ emissions, is considered a major cause of acid rain and photochemical smog formation [4]. Selective catalytic reduction (SCR) is a promising method that typically selects CO [5,6,7,8,9], H_2_ [8,9,10,11], or NH_3_ [12] as the reducing agent to eliminate emitted NO. Since CO and NO commonly coexist in exhaust gases, the catalytic reduction of NO with CO as a reducing agent can simultaneously convert CO and NO pollutants into harmless N_2_ and acceptable CO_2_. Noble metal catalysts such as Pt, Au, or Pd have been extensively studied; however, there are problems, such as high cost, low abundance, and toxicity [13,14,15,16]. Thus, it is of utmost importance to design high-efficiency and low-cost alternative catalysts to remove or reduce NO molecules.

Reducing the particle size of active metals to a few atoms is a valuable strategy to improve catalytic activity [17,18,19,20]. Compared to traditional catalysts, single-atom catalysts can greatly decrease the amount of metal used, thereby reducing costs. In particular, single-atom catalysts have been proven to efficiently catalyze or adsorb various harmful gas molecules, such as NO [21,22,23,24,25], CO [23,25], H_2_S [26], and SO_2_ [27]. Recently, a two-dimensional (2D) graphene porous material, a C_2_N monolayer, was successfully prepared via a simple wet chemical reaction [28]. This novel material with a uniform pore distribution has attracted much attention due to its large surface area and good structural stability. Given the uniform cavity structure of C_2_N, it has been demonstrated to be a suitable material for anchoring metal atoms. Previous studies have shown that metal-atom-decorated C_2_N monolayers can efficiently trap small gas molecules. For example, Ma et al. proposed 3D transition-metal-modified C_2_N as a promising candidate for the low-temperature CO oxidation reaction [29]. Anikina et al. reported that metal-decorated C_2_N monolayers have high storage capacities for H_2_ [30]. Furthermore, several studies reported that single metal-atom-anchored C_2_N monolayers can also act as effective catalysts for N_2_ reduction, oxygen reduction, and hydrogen evolution reactions [31,32,33,34,35].

The metal aluminum is environmentally friendly, inexpensive, and abundant in storage. Previous work has shown that decoration with Al atoms can significantly improve the adsorption capacity of 2D materials [36,37,38,39,40,41,42,43,44]. Specifically, Al-doped graphene showed a good adsorption ability for small molecules, such as N_2_O, NO_2_, SO_3_, and CO_2_ [36,37,38,39,40,43]. The Al-embedded C_2_N was shown to be a prospective candidate to adsorb and degrade volatile organic compounds, mainly due to a strong electronic transfer between the adsorbed molecules and Al atoms [41,42]. Strong adsorption properties of NO_2_ and NH_3_ molecules were also observed in Al-MoS_2_ [44].

Inspired by these findings, we investigated the reaction mechanism of the NO reduction with CO molecules on an Al-C_2_N monolayer based on density functional theory (DFT) calculations. The geometries of reactants, transition states, and products, as well as reaction energies, were calculated in detail. The key intermediates and preferred reaction pathways for NO reduction on Al-C_2_N were further identified. The catalytic reactivity of Al-C_2_N was also compared with other catalytic materials to evaluate the possibility of using Al-C_2_N as a catalyst for NO reduction.

## 2. Computational Methods

All DFT calculations were carried out at the level of the B3LYP exchange–correlation functional with Grimme’s DFT-D3 empirical dispersion correction using the Gaussian09 software package [45,46,47,48]. Previous literature confirmed that the B3LYP functional with DFT-D3 is a reasonable condition for calculating intermolecular non-covalent interactions [49]. The 6-31G(d, p) basis set was used to describe all atoms [50]. A pristine C_2_N cluster model in this study contained 37 carbon atoms, 12 nitrogen atoms, and 12 hydrogen atoms. All the energies were corrected with zero-point vibrational energy (ZPE). Vibration frequency calculations were performed to verify the optimized structure, where the minimum structure had no imaginary frequency, and the transition state only had one imaginary frequency. Intrinsic reaction coordinate (IRC) calculations were used to verify the transition states [51,52,53]. Atomic charges were discussed with the natural bond orbital (NBO) analysis [54]. Electron density difference (EDD) plots were obtained with the Multiwfn program [55]. For each adsorption configuration, the EDD plots were calculated as:

(1) $$\Delta \rho =\rho_{A/S}-\rho_{A}-\rho_{S}$$

where *ρ_A_*_/*S*_, *ρ_A_*, and *ρ_S_* are the electron density of the total complexes, isolated substrate, and isolated adsorbate, respectively.

The adsorption energy (E_ads_) of a given adsorbate was defined as:

(2) $$E_{\mathrm{ads}}{=E}_{\mathrm{tota}}{}_{l}-E_{A}-E_{S}$$

where E_total_, E_A_, and E_S_ are the energies of the total adsorbate-substrate systems, isolated adsorbate, and isolated substrate, respectively.

The change in Gibbs free energy (ΔG) was defined as:

(3) ΔG=ΔH−TΔS

where ΔH and ΔS represent the enthalpy with a zero-point energy correction and the entropy change at 298.15 K, respectively.

## 3. Results and Discussion

### 3.1. Geometrical Structures and Stability of Pristine C_2_N and Al-C_2_N Monolayer

**Figure 1:**
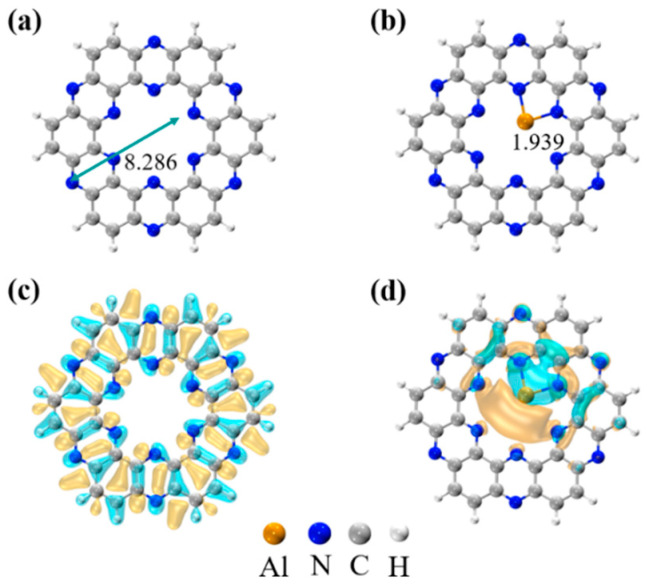
Optimized structures of the (**a**) pristine C_2_N cluster and (**b**) Al-C_2_N monolayer, respectively. Electron density difference plots (in 0.002 au) for (**c**) pristine C_2_N and (**d**) Al-C_2_N, respectively. Blue and yellow parts represent charge accumulation and depletion, respectively. All bond lengths are in Å.

Firstly, we examined the geometric structure and stability of the designed Al-C_2_N. The optimized structure of the pristine C_2_N cluster is shown in Figure 1a. The calculated lattice parameter of 8.286 Å was consistent with the experiment result (8.30 Å) [28]. Then, a single Al atom was attached to the C_2_N cluster via two adjacent N atoms (shown in Figure 1b), with both bond lengths being 1.939 Å, in which the Al atom was more preferably anchored at the corners of the six-fold cavity of the C_2_N. The calculated bond length value was in line with the previously periodic system-reported results (1.96 Å) [56]. EDD plots revealed a sizeable interaction area between the Al atom and its two adjacent N atoms. It is worth mentioning that the modification of the Al atoms could effectively change the surface properties of the C_2_N monolayer. As shown in Figure 1c,d, the uniformly distributed charge on the C_2_N monolayer changed to a directional concentrated distribution, which was essential for the subsequent adsorption of gas molecules.

To evaluate the thermal stability of the designed Al-C_2_N systems, we carried out MD simulations at 300 K and 500 K for 8 ps with a time step of 2 fs under the NVT ensemble (see Figure S1 in the Supplementary Materials). According to the MD simulations, the energies of the Al-C_2_N system fluctuated gently, suggesting its high thermodynamic stability.

### 3.2. Adsorption Behavior of NO and CO Molecules on Al-C_2_N Surface

**Figure 2:**
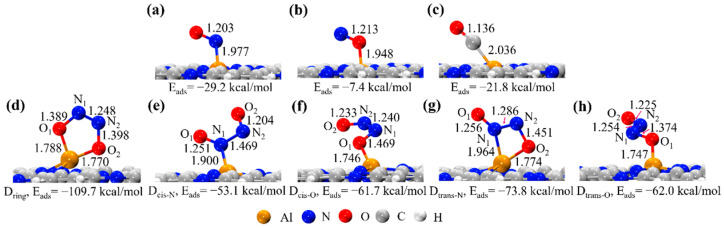
Optimized adsorption configurations for the (**a**) NO (N-end), (**b**) NO (O-end), (**c**) CO, (**d**) ring-(NO)_2_ dimers, (**e**) cis-(NO)_2_ dimers (N-end), (**f**) cis-(NO)_2_ dimers (O-end), (**g**) trans-(NO)_2_ dimers (N-end), and (**h**) trans-(NO)_2_ dimers (O-end) on Al-C_2_N surface. All bond lengths are in Å.

The stable configurations of CO and NO adsorbed on the Al-C_2_N surface are displayed in Figure 2. Table 1 summarizes the corresponding adsorption parameters for the NO and CO molecules, including the E_ads_, ΔG, and charge transfer values. Note that all the calculated ΔG values of the CO or NO molecules adsorbed on the Al-C_2_N surface were negative, suggesting that the adsorption of these species was thermodynamically spontaneous.

The adsorption geometries of the NO, CO, and (NO)_2_ dimers on Al-C_2_N are shown in Figure 2. For the NO molecules, two possible adsorption modes (including N-end and O-end) were investigated. From Figure 2a,b, it can be seen that the NO molecules were tilted concerning the Al-C_2_N surface, consistent with previous reports [24,57,58]. As evident, the calculated N-O bond lengths of the NO molecules were elongated to 1.203 Å and 1.213 Å, respectively, when compared with the free NO molecule (1.160 Å). The E_ads_ values for the N-end and O-end adsorption modes were −29.2 and −7.4 kcal/mol, respectively, which was more negative than the values in Si-doped graphene (−18.4 and −4.4 kcal/mol) [57]. From the viewpoint of adsorption energy, it is clear that the N-end adsorption was energetically more favorable than the O-end. This result was also supported by the NBO charge analysis, in which the N-end mode was accompanied by a larger charge transfer of 0.430 e from the Al-C_2_N surface to the 2π* orbital of the NO molecule (Table 1).

As for the CO molecules, our results demonstrated that CO preferred to adsorb on the Al-C_2_N surface via its C-end. Figure 2c demonstrates that the C-O bond length of CO was nearly unchanged compared to that of the free CO molecule (1.14 Å), indicating that CO was not activated after being adsorbed on the Al-C_2_N surface. Based on the E_ads_ value, the adsorption of CO (−21.8 kcal/mol) on Al-C_2_N was weaker than that of NO (−29.2 kcal/mol). In this case, it was expected that the tendency of the NO molecule to adsorb onto the Al-C_2_N surface was greater than that of CO. Unlike the NO molecules, CO acted as the electron donor, where the charge value transferred from the CO molecule to the Al-C_2_N surface was 0.083 e (Table 1).

Next, we considered the (NO)_2_ dimer configuration formed by two NO molecules co-adsorbed on the Al-C_2_N surface. The (NO)_2_ dimer was characterized for the first time by Dinerman and Ewing using infrared spectroscopy [59]. The stable (NO)_2_ dimer adsorption configurations are illustrated in Figure 2d–h. The IR spectra plots of five (NO)_2_ dimers on the Al-C_2_N surface are displayed in Figure S2. As can be seen, five different (NO)_2_ dimers were obtained on the Al-C_2_N surface.

Figure 2d shows a five-membered ring (NO)_2_ dimer structure (labeled as D_ring_), in which both NO molecules were bound to the Al site through their O-end. The bond lengths of the two formed Al-O bonds and the N_1_-N_2_ bond were 1.788, 1.770, and 1.248 Å, respectively. This structure was similar to that of Si-doped graphene (1.783, 1.762, and 1.240 Å for two Si-O bonds and the N_1_-N_2_ bond) [60]. Figure 2e,g display the cis- and trans-(NO)_2_ dimer structures at the N-end (labeled as D_cis-N_ and D_trans-N_), respectively, in which one NO molecule was adsorbed into the Al site via its N-end and two NO molecules were bound through N-N bonds. The calculated bond lengths of the N_1_-N_2_ bond were 1.469 and 1.286 Å, respectively. It is noteworthy that two novel (NO)_2_ dimer structures were explored in this work, which have not been reported in current catalysts [57,59,61,62,63,64]. Figure 2f,h correspond to two novel cis- and trans-(NO)_2_ dimer structures at the O-end (labeled as D_cis-O_ and D_trans-O_) with N_1_-N_2_ bond lengths of 1.240 and 1.254 Å, respectively. Among the above (NO)_2_ dimers, the calculated N_1_-N_2_ bond lengths ranged from 1.505 to 1.233 Å, which were much shorter than the value in the gas phase (NO)_2_ dimer (1.970 Å). As shown in Table 1, the calculated adsorption energies of the five (NO)_2_ dimers on the Al-C_2_N surface were significantly enhanced, with values of −109.7, −53.1, −61.7, −73.8, and −62.0 kcal/mol, respectively, which were larger than twice that of a NO molecule (−29.2 kcal/mol). This indicated that the addition of the second NO molecule was beneficial for strengthening the interaction between the catalyst and NO molecule. Similar results were further verified with the NBO charge analysis, where the considerable charge-transfer values from the Al-C_2_N surface to (NO)_2_ dimers were −1.377, −0.672, −0.731, −1.303, and −0697, respectively (Table 1).

### 3.3. NO Reduction Mechanism on Al-C_2_N Surface

Here, the NO reduction mechanism mainly included the direct decomposition process and the (NO)_2_ dimer reduction process. For the former, a NO molecule was directly decomposed into O and N atoms. For the latter, two NO molecules were co-adsorbed forming (NO)_2_ dimers, followed by their decomposition into N_2_O molecules and O atoms. Subsequently, the N_2_O molecules were desorbed, and the remaining O atoms could be removed with the NO or CO molecules.

#### 3.3.1. NO Direct Decomposition Process

**Figure 3:**
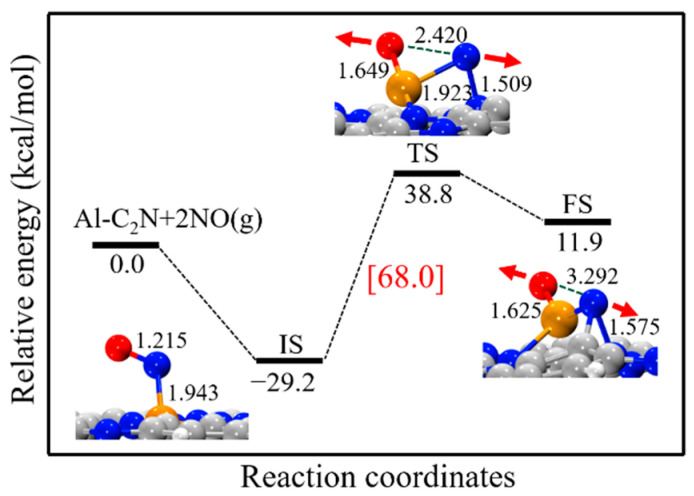
The energy profile and corresponding structure for the NO direct decomposition process on Al-C_2_N. All bond lengths are in Å.

Figure 3 shows the energy profile of the NO direct decomposition process on Al-C_2_N, where the energy sum of Al-C_2_N and free NO molecules was set as the reference energy. As seen, the reaction began with the NO molecule adsorbed on Al-C_2_N via its N-end. In the TS structure, the calculated O-N bond length of the NO molecule was elongated from 1.215 Å to 2.420 Å. In the FS structure, the O-N bond was broken and the distance between the O and N atoms was 3.292 Å. Our results showed that the NO direct decomposition process was unfavorable both in kinetics and thermodynamics due to the high reaction energy barrier (68.0 kcal/mol) and endothermic nature (41.1 kcal/mol), which agreed withprevious reports, such as Si-doped graphene (39.2 kcal/mol) [24], Si-doped BN nanosheets (57.9 kcal/mol) [60], and Fe-doped graphene (124.1 kcal/mol) [25].

#### 3.3.2. (NO)_2_ Dimer Reduction Process

In this section, we examined the possible reaction pathways of the (NO)_2_ dimer reduction process on Al-C_2_N. There were four reaction pathways starting with different (NO)_2_ dimer structures described as path I, path II, path III, and path IV, respectively. For simplicity, the remaining oxygen atoms on the Al-C_2_N surface were labeled as O_ads_.

**Figure 4:**
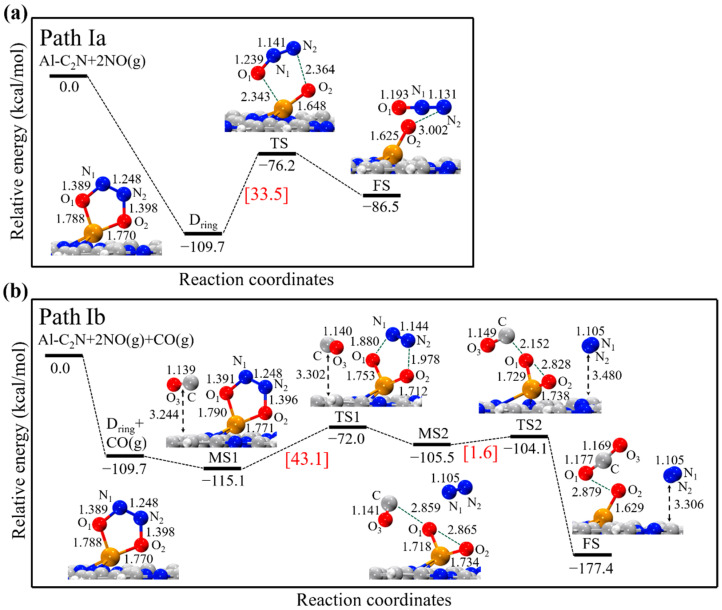
The energy profile and corresponding structure of the D_ring_ dimer reduction process on Al-C_2_N; (**a**) (NO)_2_ → N_2_O + O_ads_ (path Ia), (**b**) (NO)_2_ + CO → N_2_ + CO_2_ + O_ads_ (path Ib). All bond lengths are in Å.

In path Ia, the five-membered ring (NO)_2_ dimer structure (D_ring_) was the initial state. The energy profile and corresponding minima state and transition state are displayed in Figure 4a. As can be seen, the D_ring_ structure could be decomposed into the product (N_2_O + O_ads_) through the transition state with a high-energy barrier of 33.5 kcal/mol. In the TS structure, the N_2_-O_2_ bond broke with the bond length increasing from 1.398 to 2.364 Å, while the N_1_-N_2_ bond length decreased from 1.248 to 1.141 Å. The entire process from D_ring_ to the FS structure was endothermic by 23.2 kcal/mol. Given the high reaction barrier and endothermicity, it was expected that the D_ring_ dimer reduction on Al-C_2_N was unfavorable both kinetically and thermodynamically.

In path Ib, a two-step reaction was identified: (i) (NO)_2_ → N_2_ + 2O_ads_, followed by (ii) CO + O_ads_ → CO_2_. As shown in Figure 4b, the D_ring_ structure was taken as the initial state and, subsequently, CO was physisorbed over Al-C_2_N to form an intermediate state (the MS1 structure). In the TS1 structure, two N-O bonds broke with the bond lengths increasing to 1.880 and 1.978 Å, respectively, while the N_1_-N_2_ bond length was shortened to 1.144 Å. Next, N_2_ was completely formed in the MS2 structure. In the next step, CO approached the O_1_ atom. The O_1_···C bond’s length reduced from 2.859 to 2.152 Å and, finally, formed the CO_2_ molecule. Note that the energy barriers of the first and second steps were 43.1 and 1.6 kcal/mol, respectively, which could be provided by the larger exothermic reaction energy (−67.7 kcal/mol, from D_ring_ to FS).

**Figure 5:**
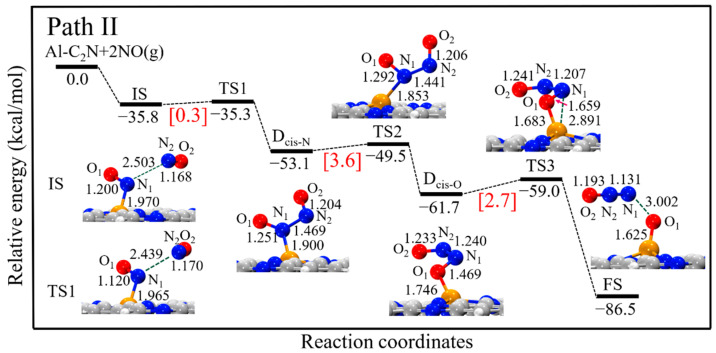
The energy profiles and corresponding structures of the D_cis-N_ and D_cis-O_ dimer reduction process on Al-C_2_N. All bond lengths are in Å.

In path II, the reaction started with the co-adsorption of two NO molecules to generate a cis-(NO)_2_ dimer (N-end, D_cis-N_) structure, as shown in Figure 5. As seen, this step had a negligible energy barrier and was exothermic by 17.3 kcal/mol. Then, the D_cis-N_ structure could be converted to the more stable cis-(NO)_2_ dimer (O-end, D_cis-O_) structure by overcoming a small energy barrier of 3.6 kcal/mol, being exothermic by 8.6 kcal/mol. Finally, the D_cis-O_ structure decomposed into the product (N_2_O and O_ads_ species) through TS2 by breaking the N_1_-O1 bond. In the TS2 structure, the N_1_-O_1_ distance significantly elongated from 1.469 to 1.659 Å, while the N_1_-N_2_ distance decreased from 1.240 to 1.207 Å. We note that there was a negligible energy barrier for this step (2.7 kcal/mol), which was exothermic by 24.8 kcal/mol. Since the entire reaction was a highly exothermic process (−50.7 kcal/mol, from IS to FS), it was thermodynamically feasible under mild conditions.

**Figure 6:**
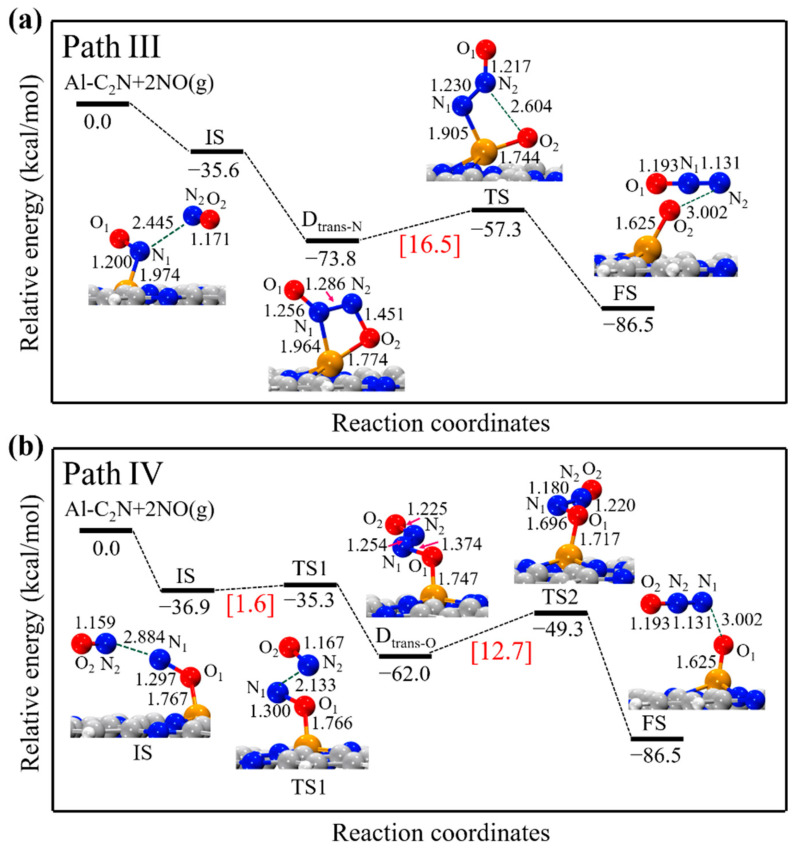
The energy profiles and corresponding structures of the (**a**) D_trans-N_ and (**b**) D_trans-O_ dimer reduction process on Al-C_2_N. All bond lengths are in Å.

In path III, the trans-(NO)_2_ dimer structure (N-end, D_trans-N_) was considered the starting point for the NO reduction on Al-C_2_N. From Figure 6a, one could see that the NO molecule bonded with the Al site through the N-end, whereas another NO molecule was weakly physisorbed on the surface, with the distance between the N_1_ and N_2_ atoms being 2.445 Å. The co-adsorption energy of 2NO was −35.6 kcal/mol. Next, the D_trans-N_ structure was formed through a barrierless process. In this structure, the calculated N_1_-N_2_ bond was shortened to 1.286 Å, while the N_2_-O_2_ bond was extended to 1.451 Å. In the TS structure, the N_2_-O_2_ bond was significantly extended from 1.451 to 2.604 Å. Finally, the N_2_-O_2_ bond was completely broken, forming N_2_O and O_ads_ moieties. This path revealed a high reaction barrier of 16.5 kcal/mol and was exothermic by 12.7 kcal/mol. Figure 6b exhibits path IV, starting from the trans-(NO)_2_ dimer structure (O-end, D_trans-O_). In this path, 2NO molecules formed the D_trans-O_ structure through an extremely low-energy barrier (1.6 kcal/mol). Then, the N_1_-O_1_ bond length was significantly extended from 1.374 Å in the D_trans-O_ structure to 1.696 Å in the TS2 structure. The energy barrier for this step was 12.7 kcal/mol, which could be provided by the exothermic reaction energy (−24.5 kcal/mol).

According to our results, it was found that the NO reduction preferred to proceed via the (NO)_2_ dimer reduction process. First, the E_ads_ values of the (NO)_2_ dimers were much larger than that of the single NO molecule. Second, the (NO)_2_ dimer reduction process was thermodynamically and kinetically more favorable than the NO direct decomposition process. Based on the energy barriers (E_a_) and reaction energies (ΔE_r_), the NO dimer reduction on the Al-C_2_N surface could occur via path II and path IV (Table 1). Path II was energetically the most favorable pathway with the max energy barrier for the (NO)_2_ → N_2_O + O_ads_ reaction of only 3.6 kcal/mol, which was even smaller than the values in noble metal catalysts, such as Pd-BNNS (14.9 kcal/mol) [58], Au (8.1 kcal/mol) [65], and Ag (6.2 kcal/mol) [66]. These results implied that the Al-C_2_N catalyst exhibited good catalytic activity towards the NO reduction.

**Figure 7:**
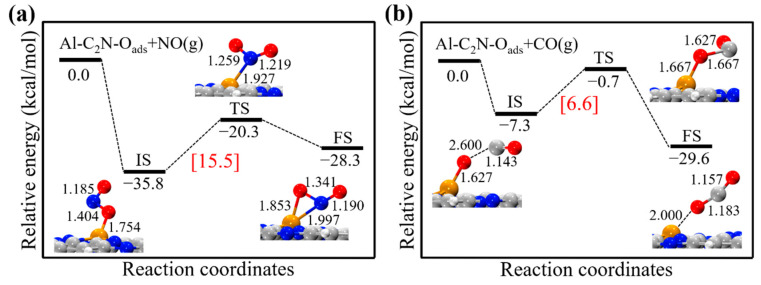
The energy profiles and corresponding structures for the step of (**a**) O_ads_ + NO → NO_2_ and (**b**) O_ads_ + CO → CO_2_ on Al-C_2_N, respectively. All bond lengths are in Å.

After the N_2_O desorption, the remaining O_ads_ atom could be removed with the NO or CO molecules. In our previous work, we revealed that Al-C_2_N could serve as a promising catalyst for N_2_O reduction to environmentally friendly N_2_ molecules [67]. Figure 7 shows the reaction pathways of O_ads_ + NO → NO_2_ and O_ads_ + CO → CO_2_ on Al-C_2_N, respectively. Our results showed that O_ads_ + NO → NO_2_ was an endothermic process (7.5 kcal/mol), and quite a high-energy barrier (15.5 kcal/mol) required to be surmounted. As seen in Figure 7b, the O_ads_ + CO → CO_2_ reaction was an exothermic process, and an energy barrier of only 6.6 kcal/mol was needed for Al-C_2_N, which was smaller than the value for Pt-graphene (13.4 kcal/mol) [68]. This meant that CO_2_ molecules were more likely to form on the Al-C_2_N catalyst in the existence of NO molecules.

## 4. Conclusions

In this work, we investigated the NO reduction over low-cost Al-C_2_N catalysts using DFT calculations in detail. According to the adsorption energy and charge transfer values, the adsorption of NO on the catalyst was significantly stronger than that of CO, which suggested that the Al-C_2_N catalyst was more selective to NO than CO. For the NO reduction mechanism, our results showed that the NO direct decomposition process was barely possible due to the extremely high-energy barrier and endothermicity. In contrast, the catalysis of the NO reduction via the (NO)_2_ dimer reduction process was both thermodynamically and kinetically favorable. It was found that cis-(NO)_2_ dimer structures were key intermediates for the NO reduction, where the calculated max barriers along the most energetically favorable pathway (path II) was only 3.6 kcal/mol. The remaining O_ads_ species on Al-C_2_N could be eliminated with CO molecules, which required overcoming the energy barriers of only 6.6 kcal/mol. Overall, Al-C_2_N is expected to be a promising catalyst for NO reduction with CO.

## Figures and Tables

**Table 1:** Calculated adsorption energies (E_ads_, kcal/mol), adsorption free energies (∆G, kcal/mol), and net charge-transfer values (q, e) for different adsorption species on the Al-C_2_N surface, along with the corresponding energy barriers (E_a_, kcal/mol) and reaction energies (ΔE_r_) for a single NO or (NO)_2_ dimer reduction on Al-C_2_N surface.

Adsorbate	E_ads_	∆G	q ^1^	E_a_	Path	ΔE_r_
CO	−21.8	−11.4	0.083	-	-	-
NO (O-end)	−7.4	−4.1	−0.337	-	-	-
NO (N-end)	−29.2	−17.8	−0.430	68.0	-	41.1
D_ring_	−109.7	−86.3	−1.377	33.5 (43.1)	Ia (Ib)	23.2 (−67.7)
D_cis-N_	−53.1	−31.8	−0.672	3.6	II	−8.6
D_cis-O_	−61.7	−39.9	−0.731	2.7	II	−24.8
D_trans-N_	−73.8	−52.3	−1.303	16.5	III	−12.7
D_trans-O_	−62.0	−40.0	−0697	12.7	IV	−24.5

^1^ Positive and negative values of q correspond to the net charge transfer from the adsorbate to the Al-C_2_N and the net charge transfer from the Al-C_2_N surface to the adsorbate, respectively.

## Data Availability

All data presented in this study are available in this published article and Supplementary Materials.

## Supplementary Materials

The following supporting information can be downloaded at: https://www.mdpi.com/article/10.3390/molecules27185790/s1. Figure S1: molecular dynamics simulation for Al-C_2_N catalyst at (a) 300 K and (b) 500 K, respectively; Figure S2: IR spectra plots for five kinds of (NO)_2_ dimers on the Al-C_2_N surface.

## Sample Availability

Samples of the compounds are not available from the authors.
