# Supplementary material for: Al-Decorated C2N Monolayer as a Potential Catalyst for NO Reduction with CO Molecules: A DFT Investigation
Source: Molecules. 2022 Sep 7;27(18):5790. doi: 10.3390/molecules27185790 (PMC9503404; doi:10.3390/molecules27185790)
Supplement: Supplementary file 1 [file molecules-27-05790-s001.zip › molecules-1860527-supplementary.pdf]

molecules

MDPI

Supplementary Materials: Al-Decorated C_2N Monolayer as a Potential Catalyst for NO Reduction by CO Molecule: A DFT Investigation

Xinmiao Liu †, Yunjie Xu † and Li Sheng *

MIIT Key Laboratory of Critical Materials Technology for New Energy Conversion and Storage, School of Chemistry and Chemical Engineering, Harbin Institute of Technology, Harbin 150001, China
* Correspondence: shengli@hit.edu.cn
† These authors contributed equally to this work.

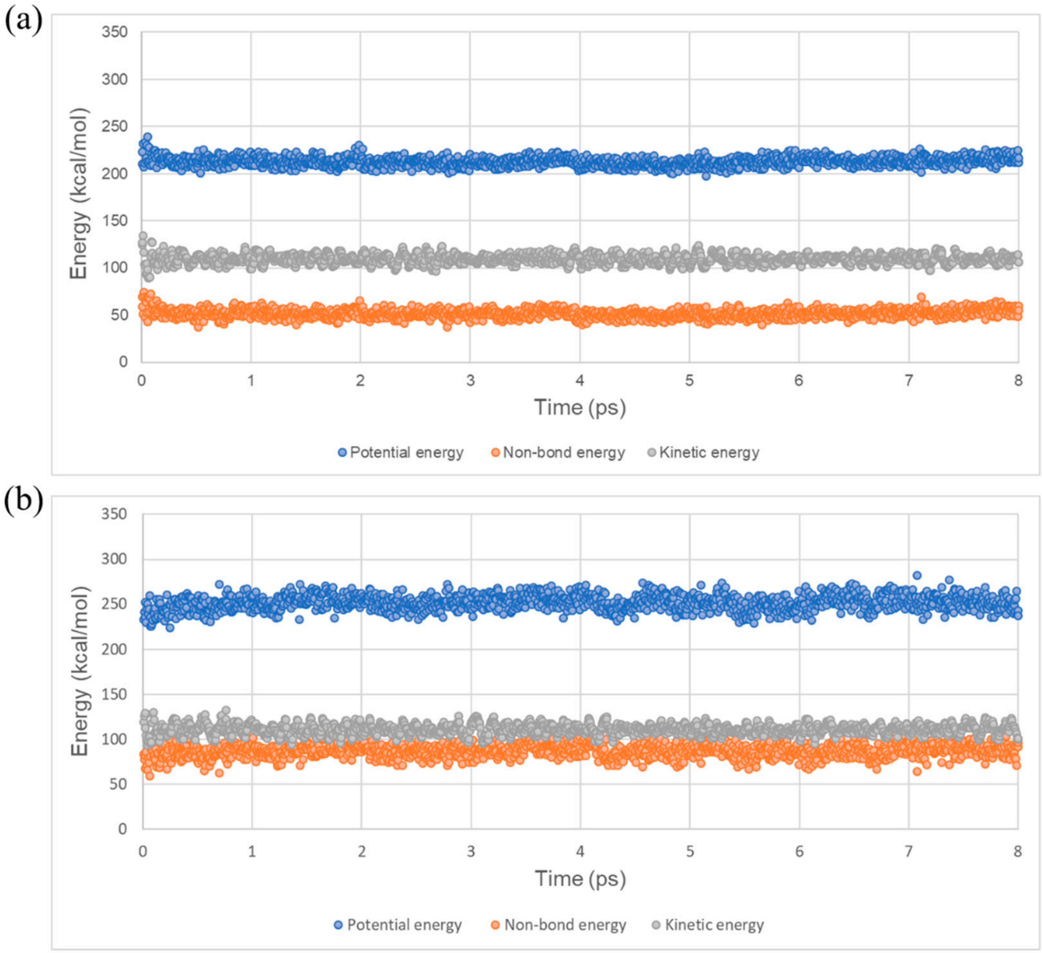

Figure S1. Molecular dynamics simulation for Al-C_2N catalyst at (**a**) 300 K and (**b**) 500 K, respectively.

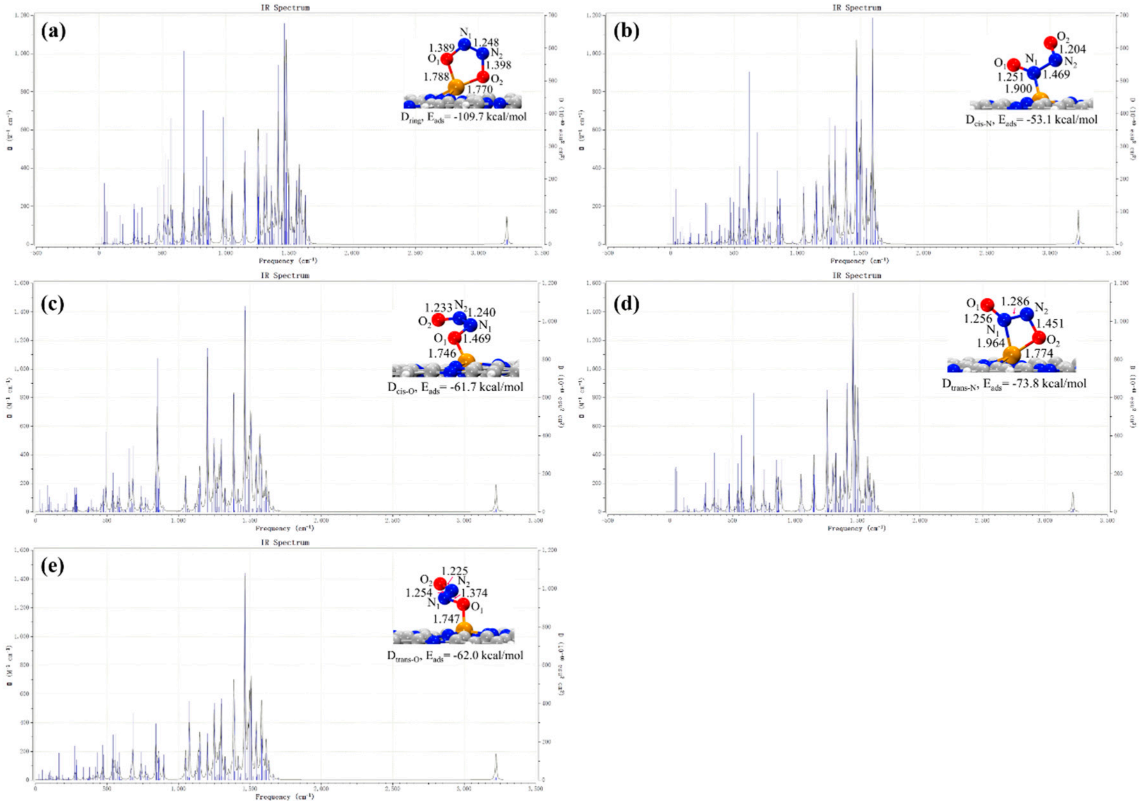

Figure S2. IR spectra plots for five kinds of $(NO)_2$ dimers on the Al-C_2N surface.
